# Why Black Flowers? An Extreme Environment and Molecular Perspective of Black Color Accumulation in the Ornamental and Food Crops

**DOI:** 10.3389/fpls.2022.885176

**Published:** 2022-04-14

**Authors:** Sagheer Ahmad, Jinliao Chen, Guizhen Chen, Jie Huang, Yuzhen Zhou, Kai Zhao, Siren Lan, Zhongjian Liu, Donghui Peng

**Affiliations:** ^1^Key Laboratory of National Forestry and Grassland Administration for Orchid Conservation and Utilization at College of Landscape Architecture, Fujian Agriculture and Forestry University, Fuzhou, China; ^2^College of Life Sciences, Fujian Normal University, Fuzhou, China

**Keywords:** heat stress, black flower color, anthocyanins, ornamental crops, pollination

## Abstract

Pollinators are attracted to vibrant flower colors. That is why flower color is the key agent to allow successful fruit set in food or ornamental crops. However, black flower color is the least attractive to pollinators, although a number of plant species produce black flowers. Cyanidin-based anthocyanins are thought to be the key agents to induce black color in the ornamental and fruit crops. R2R3-MYB transcription factors (TFs) play key roles for the tissue-specific accumulation of anthocyanin. MYB1 and MYB11 are the key TFs regulating the expression of anthocyanin biosynthesis genes for black color accumulation. Post-transcriptional silencing of *flavone synthase II* (*FNS*) gene is the technological method to stimulate the accumulation of cyanidin-based anthocyanins in black cultivars. Type 1 promoter of *DvIVS* takes the advantage of *FNS* silencing to produce large amounts of black anthocyanins. Exogenous ethylene application triggers anthocyanin accumulation in the fruit skin at ripening. Environment cues have been the pivotal regulators to allow differential accumulation of anthocyanins to regulate black color. Heat stress is one of the most important environmental stimulus that regulates concentration gradient of anthocyanins in various plant parts, thereby affecting the color pattern of flowers. Stability of black anthocyanins in the extreme environments can save the damage, especially in fruits, caused by abiotic stress. White flowers without anthocyanin face more damages from abiotic stress than dark color flowers. The intensity and pattern of flower color accumulation determine the overall fruit set, thereby controlling crop yield and human food needs. This review paper presents comprehensive knowledge of black flower regulation as affected by high temperature stress, and the molecular regulators of anthocyanin for black color in ornamental and food crops. It also discusses the black color-pollination interaction pattern affected by heat stress for food and ornamental crops.

## Introduction

Flower color is one of the most conspicuous attributes of angiosperms. Since antiquity, flower petal color has been the key to pollinator attraction. Although most of the angiosperms produce vibrant color flowers that are more attractive to pollinators, a few species generate black color in the flowers. Apparently, the black color is not much attractive to the pollinators, but it is not out of need. Both plants and pollinators are benefitted by black color. Heat stress and high temperature are closely associated with black color impacts on plants, pollinators, and pollination strategies. The most prevalent pigments to paint flowers black are the anthocyanins.

Anthocyanins are widely distributed in nature (Wu and Prior, 2005) and give attractive colors to flowers, grains, and fruits (Kong et al., 2003; Escribano-Bailón et al., 2004). Anthocyanins are important chemotaxonomic and quality indicators in plants and their antioxidant ability gains much interest for health (Kong et al., 2003; Março and Scarminio, 2007; Salehi et al., 2020; Paun et al., 2022). They are helpful to cure age-induced oxidative stress, cardiovascular disorders, and inflammatory responses (Hassellund et al., 2013). It is believed that anthocyanin is synthesized at cytosolic surface of endoplasmic reticulum (ER), and it accumulates in the vacuole (Han et al., 2022). MRP (multidrug resistance-associated protein), MATE (multidrug and toxic compound extrusion), and GST (glutathione S-transferase) are mainly responsible for the transport of anthocyanin from cytoplasm to vacuole (Hu et al., 2016; Han et al., 2022).

Anthocyanins are sugar-containing equivalents (3-glucosides) of anthocyanidins (Mekapogu et al., 2020). They are water-soluble glycosides and acylglycosides derived from anthocyanidins (Wu et al., 2004). Anthocyanidins possess two aromatic benzene rings which are separated by an oxygenated heterocycle (Noda et al., 2000; Mekapogu et al., 2020). Petal color is mainly determined by the number of hydroxyl groups in the B-ring. An increase in hydroxyl groups causes color shift to blue (Noda et al., 2000). Six anthocyanidins are widely distributed in vegetables and fruits, including malvidin, petunidin, peonidin, cyanidin, delphinidin, and pelargonidin (de Pascual-Teresa et al., 2002; Di Paola-Naranjo et al., 2004; Lohachoompol et al., 2008). The most abundant anthocyanidins in flowers include pelargonidin, delphinidin, and cyanidin (Mekapogu et al., 2020). Cyanidins usually impart magenta (reddish-purple) color and delphinidins appear purple or blue-red (Yang et al., 2022). Cyanidin causes purple-red color in chrysanthemum flowers (Kawase and Tsukamoto, 1976; Noda et al., 2000). Violet transgenic flowers of chrysanthemum are due to delphinidins, such as delphinidin 3-(3″,6″-dimalonyl) glucoside and delphinidin 3-(6″-malonyl) glucoside (Noda et al., 2013).

High accumulation of cyanidin-based anthocyanins is responsible for black color in ornamental crops (Deguchi et al., 2013, 2015). Three TF families (MYBs, bHLHs, and MBW) regulate the genes involving anthocyanin biosynthesis (Zhao et al., 2013). The *PeMYB11* is the major R2R3-MYB TF that regulates the black color production (Hsu et al., 2019). The *FNS* and *IVS* are the key genes involving the biosynthesis and regulation of black anthocyanins. Non-pigmented flowers face more damages from abiotic stress than pigmented flowers. Dark color flowers get more favor in the dry conditions than light color flowers (Sullivan and Koski, 2021). High temperature upregulates the expression of most of the anthocyanin biosynthesis genes (Zhang et al., 2019). Moreover, dark petal color increases the internal flower temperature (Tikhomirov et al., 1960), attracting more pollinators during winter. Therefore, this review curtails the black color regulation by anthocyanins, the impact of black color on plant-pollinator interactions and the association of temperature fluctuations with color intensity.

## Impact of High Temperature on Anthocyanin Gradient and Color Formation

**Figure 1:**
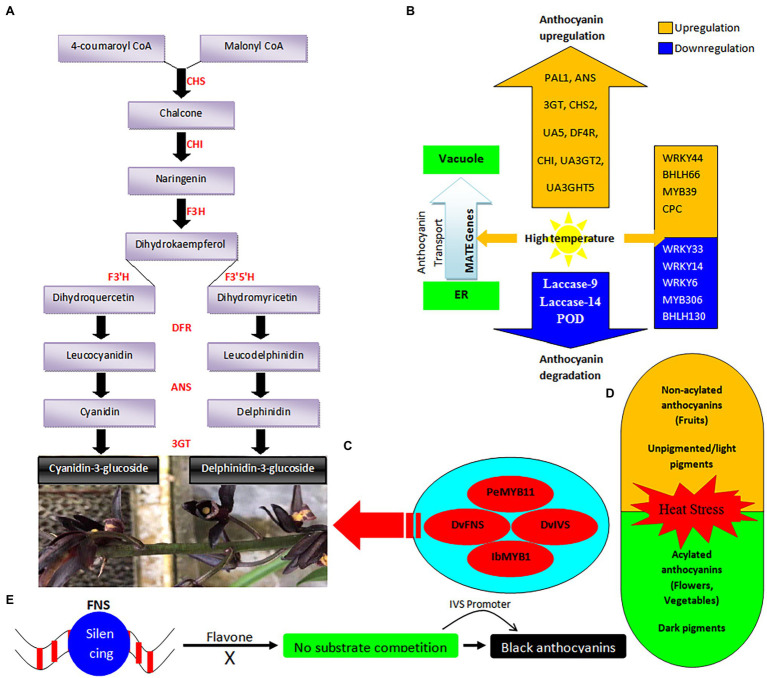
Summary of black color anthocyanin accumulation pathways **(A)**; effect of high temperature on molecular regulation of deep color anthocyanins **(B)**; the key genetic regulators of black color anthocyanin accumulation in the flower petals **(C)**; the common effects of heat stress on plant anthocyanins (orange color shows more susceptibility towards heat stress and the green color shows stability towards heat stress; **D**); and the proposed pathway of black anthocyanin generation through induced gene silencing **(E)**.

Biosynthesis of anthocyanins is affected by biotic and abiotic factors, such as nutrients, light, water stress, and temperature (Ubi, 2004). High temperature affects the biosynthesis of anthocyanins. The biosynthesis pathway of anthocyanin can be divided in two phases. The early biosynthesis is regulated by genes such as CHS (chalcone synthase), CHI (chalcone isomerase), F3H (flavanone 3-hydroxylase), F3′H (flavanone-3′-hydroxylase) and F3′5′H (flavanone-3′5′-hydroxylase). The late biosynthesis is regulated by genes such as ANS (anthocyanin synthase), DFR, and UGFT (UDP-glycose: flavonoid 3-O-glycosyltransferase; Figure 1A). High temperature (35°C) significantly upregulates the expression of PAL1, ANS, 3GT, CHS2, UA5, DF4R, CHI, UA3GT2, and UA3GHT5, causing increase in anthocyanin contents in strawberries (Zhang et al., 2019; Figure 1B). High storage temperature improves *UA3GT2*, a UDP-glucose: anthocyanidin 3-O-glucosyltransferase (UGAT) gene correlated with high-temperature induced anthocyanin accumulation (Zhang et al., 2019). Recently, *MATE TT12* is thought to involve cross-membrane anthocyanin transportation in strawberry, cotton and radish (Gao et al., 2016; Chen et al., 2018; M’mbone et al., 2018). High temperature upregulated the expression of six *MATE TT12* genes in strawberry, thereby increasing anthocyanidin levels from endoplasmic reticulum to vacuole (Figure 1B). This further deepens the fruit color. High temperature upregulates MATE genes, *MATE TT2*, *MATE DTX1*, in strawberry (Zhang et al., 2019).

However, high temperature stress also causes low accumulation of pigments in apple (Palmer et al., 2010; Lin-Wang et al., 2011), by repressing the expression of anthocyanin biosynthesis genes and enzymes (Iglesias et al., 1999; Ban et al., 2007; Palmer et al., 2010; Lin-Wang et al., 2011). Decrease in orchard temperature improves the apple skin color, suggesting that anthocyanin biosynthesis is influenced by change in temperature (Iglesias et al., 2002, 2005).

High temperature activates the expression of anthocyanin degradation genes, such as *laccase-9* and *laccase-14*, and it also stimulates the degradation of anthocyanins by enhancing the POD (Peng et al., 2017). Thus, high ambient temperature causes both accumulation and degradation of anthocyanin at the same time (Niu et al., 2017).

Heating magnitude and duration has a strong influence on the stability of anthocyanins (Patras et al., 2010). High ambient temperature may instigate loss of fresh color after harvesting, causing dark red appearance of strawberries leading to serious economic losses (Peng et al., 2017). Exposure of elderberry to 95°C caused 50% loss of anthocyanin contents (Sadilova et al., 2006). Researchers have documented that an arithmetic increase in temperature causes logarithmic anthocyanin destruction (Havlíková and Míková, 1985; Drdak and Daucik, 1990; Rhim, 2002). Exposure of blueberries to 95°C for 3 min causes 43% loss of anthocyanins (Brownmiller et al., 2008). However, anthocyanin from black carrot were comparatively stable up to 90°C (Rhim, 2002; Kırca et al., 2006), probably due to di-acylation of anthocyanin structure. Acylation protects anthocyanin from hydration, thereby making it more stable (Goto et al., 1979; Brouillard, 1981). High temperature of 95°C causes 53% degradation of cyanidin-3-glucoside in blackcurrant extract (Rubinskiene et al., 2005). Cyanidin-3-rutinoside is the most stable anthocyanin at 95°C in blackcurrant (Rubinskiene et al., 2005).

Temperature affects the expression of flower color (Dela et al., 2003). High temperature causes poor flower color in flowers, such as chrysanthemum (Stickland, 1974; Nozaki et al., 2005, 2006), carnation (Maekawa and Nakamura, 1977), petunia (Shvarts et al., 1997), rose (Shisa and Takano, 1964; Dela et al., 2003), Kangaroo Paw (Ben-Tal and King, 1997), and lily (Lai et al., 2011). High temperature (32°C) causes decrease in anthocyanin contents in petunia flowers (Shvarts et al., 1997), as anthocyanin synthesis is inhibited by elevated temperature conditions (Yamagishi et al., 2010; Lai et al., 2011).

## Heat Stress Affects Anthocyanin Biosynthesis and Accumulation

Anthocyanins significantly affect the performance of plants during abiotic stress (Rausher, 2008; Sullivan and Koski, 2021). Anthocyanin helps plants in their tolerance against abiotic stress and presents an important biological event during ripening of fruits, such as strawberry, plum, cherry, red orange, and grape (Shin et al., 2007; Flores et al., 2015; Carmona et al., 2017; Martínez-Romero et al., 2017). Anthocyanins act as photoreceptors, anti-oxidants, and osmoregulators, making plants tolerant against abiotic stress (Gould, 2004).

White flowers without anthocyanin face more damages from abiotic stress than pigmented flowers (Figure 1D). Dark color flowers get more favor in the dry conditions than light color flowers (Sullivan and Koski, 2021). Drier *Clarkia xantiana* flowers accumulate more anthocyanin contents as compared to unpigmented morphs (Warren and Mackenzie, 2001; Vaidya et al., 2018). High reproductive success of pigmented *Ipomoea purpurea* was observed under heat stress as compared to unpigmented morphs (Coberly and Rausher, 2003). An increase in global temperature may cause decline in floral pigmentation in the case if decreased pigmentation allow proper functioning of heat-sensitive reproductive parts (van der Kooi et al., 2019). However, increased drought conditions may support pigmented morphs (Warren and Mackenzie, 2001).

The most important issue of natural colorants is their low stability under high temperature (Oancea, 2021). However, anthocyanins are comparatively more stable to heat stress (Albuquerque et al., 2021). The main anthocyanin structure contains 2-phenylbenzopyrylium heterocycle C-15 skeleton called anthocyanidin or aglycon. This skeleton contains ▬OCH3 or ▬OH groups (Oancea, 2021). Presence of ▬OH group reduces stability and increases blue color, while the presence of ▬OCH3 groups elevates stability and redness (Albuquerque et al., 2021). Changes in the structure of anthocyanins is caused by fluctuation in the number of ▬OH groups, intensity of methylation of ▬OH groups, the number, and nature of attached sugar moiety to the phenolic molecules (McGhie and Walton, 2007; Patras et al., 2010). Degradation is mainly caused by breakage of covalent bonds, oxidation or heat-triggered increase of oxidation reactions. Opening of pyrylium ring and the formation of chalcone is the first step of anthocyanin degradation stimulated by heat stress (Palamidis and Markakis, 1975; Patras et al., 2010). Upon heating, the anthocyanin decomposes into chalcone structure (Adams, 1973).

Increasing temperature negatively affects the stability of cyanidin-3-O-rutinoside and cyanidin-3-O-glucoside in black rice (Sui et al., 2014). Some anthocyanins, such as pelargonidin-3-O-glucoside and cyanidin-3-O-glucoside from strawberries and blackberries, are more susceptible to heat (Shahidi, 2012). However, the methoxylation and acylation increase anthocyanin stability against heat stress (Shahidi, 2012). Acylated anthocyanins are generated after the acylation of glycosyl groups of anthocyanins with organic acids, thereby increasing heat stability. Diacylated anthocyanins provide significantly high blue color stability to red cabbage at 50°C as compared to non-acylated anthocyanins (Fenger et al., 2020). Thus, acylation of anthocyanins is essential in technological applications to produce colorants with prolonged half-life. However, extreme heat stress (95°C) causes decomposition of acylated anthocyanins in black carrot (Sadilova et al., 2007). Acylated anthocyanins are present in flowers and vegetables, while non-acylated anthocyanins are mostly distributed in fruits (Vidana Gamage et al., 2021; Figure 1D). In black carrot, acylated anthocyanins remain stable to temperature increase of 20–50°C than non-acylated anthocyanins from blackberry (Zozio et al., 2011).

Heat stress increases the anthocyanin contents of purple wheat (De Leonardis et al., 2015; Li et al., 2018). Increased accumulation of anthocyanins was stimulated by upregulation of drought stress-related genes (Castellarin et al., 2007; Cui et al., 2017; Massonnet et al., 2017). Under water drought conditions, higher contents of anthocyanins were observed in apple (Kilili et al., 1996; Mills et al., 1996), strawberry (Ikeda et al., 2011; Rugienius et al., 2015), pomegranate (Laribi et al., 2013) and apricot (Torrecillas et al., 2000; Pérez-Pastor et al., 2007). Increase in temperature increases anthocyanin contents of dark red jujube (Jiang et al., 2020). However, draught also negatively affects anthocyanin accumulation due to reduced photosynthesis, causing poor color development (Bahar et al., 2011).

Anthocyanin degradation due to high temperature causes gradual color loss of *Malus profusion* fruits in summer (Rehman et al., 2017). High temperature treatment of more than 33°C significantly reduced the concentration of cyanidin 3-galactoside. This reduction is caused by the downregulation of anthocyanin biosynthesis genes (*MpUFGT*, *MpDFR*, *MpLDOX*, *MpCHS*, and *MpMYB10*; Steyn et al., 2004; Ubi et al., 2006; Rehman et al., 2017). High temperature also stimulated the generation of H_2_O_2_ by enhancing the activities of MDA, SOD, and cell sap pH (Rehman et al., 2017). Moreover, the expression of anthocyanin transport genes (*MpVHA-B1* and *MpVHA-B2*) was also reduced.

## Transcription Factors for Anthocyanin Formation and Color Schemes

Anthocyanin biosynthesis is regulated by a number of transcription factor families, including MYB (v-myb avian myeloblastosis viral oncogene homolog), bHLH (basic helix–loop–helix), WRKY, CPC, and WD40 (WD40-repeats proteins; Herrera Valderrama et al., 2014; Bai et al., 2017). R2R3-MYB TFs play key roles in providing the specificity for the downstream genes, causing tissue-specific accumulation of anthocyanin (Koes et al., 2005; Feller et al., 2011; Hichri et al., 2011; Petroni and Tonelli, 2011). The bHLH TFs essentially regulate the activity of R2R3-MYB partner by promoting its transcription or stabilizing the protein complexes (Hernandez et al., 2004). The WDR proteins physically interact with bHLH and MYB TFs to regulate the biosynthesis of anthocyanins (Zhang et al., 2003). Thus, MBW (MYB, bHLH, and WD40) complex primarily regulates anthocyanin biosynthesis genes (Gonzalez et al., 2008; Petroni and Tonelli, 2011). Most of the MYBs are positive regulators of anthocyanin biosynthesis (Jaakola, 2013). However, some MYBs repress it too, such as grapevine VvMYB4 and strawberry FaMYB1 and FaMYB9 (Schaart et al., 2013).

In the *Phalaenopsis* cultivar ‘Panda’, MYB TFs *PeMYB7*, *PeMYB11*, and miRNA156g and miR858 are responsible for purple spot formation in sepals (Zhao et al., 2019). The *PeMYB11* is the major R2R3-MYB TF that regulates the black color production (Hsu et al., 2019; Figure 1C). A retrotransposon HORT1 (Harlequin Orchid RetroTransposon 1) causes very strong expression of *PeMYB11*, leading to extremely high anthocyanin accumulation in the harlequin flowers of *Phalaenopsis* (Hsu et al., 2019; Zhao et al., 2019). The miR156 and miR858 are the key interference RNAs for PeMYB7 and PeMYB11 (Zhao et al., 2019). High expression of anthocyanin biosynthesis pathway genes (*PeCHI*, *PeANS*, *PeC4H*, *PeF3H*, *PeF3’H*, *Pe3HI*, and *Pe4CL2*) was observed in spot tissues as compared to non-spot tissues (Figure 1B). Moreover, the ectopic *MYB* or *bHLH* expression causes dark purple color in transgenic plants, such as *Leaf Color* (bHLH) and *Deep Purple* (MYB) from petunia (Albert et al., 2009, 2011).

The black color formation has been studied in a few fruits and vegetables, such as purple cauliflower (*Brassica oleracea* L. var. *botrytis*; Chiu et al., 2010), purple sweet potato (*Ipomoea batatas*; Mano et al., 2007), and blood oranges (*Citrus sinensis*; Butelli et al., 2012). In blood oranges, insertion of a *Copia*-like retrotransposon in the upstream region of a R3R3-MYB TF gene, *Ruby*, causes extreme accumulation of anthocyanin in the fruit (Butelli et al., 2012). In the purple cauliflower, insertion of a *Harbinger* DNA transposon in the regulatory region of a R2R3-MYB TF encoding gene, *Purple* (*Pr*), causes upregulation of *Pr*, resulting in dark color accumulation (Chiu et al., 2010). Sweet potato purple color is caused by predominant expression of *IbMYB1* (Mano et al., 2007). Storage of strawberries at high temperature upregulated *WRKY44*, *bHLH128*, *bHLH66*, *MYB39*, and *CPC*, and downregulated *WRKY33*, *WRKY14*, *WRKY6*, *MYB306*, and *bHLH130* (Zhang et al., 2019; Figure 1B).

Therefore, high expression of the regulatory TFs in the biosynthesis pathway of anthocyanin may cause black flowers and fruits in plants. However, the detailed molecular mechanisms are yet to be elucidated.

## Role of Hormones in Deep Coloration

Phytohormones, such as cytokinin, abscisic acid, jasmonate, and ethylene, play significant roles in color development through increasing anthocyanin accumulation (Jia et al., 2011; Colquhoun et al., 2012; Merchante et al., 2013). However, gibberellins and auxins reduce the biosynthesis of anthocyanins during fruit color development (Jaakola, 2013). Ethylene is the key hormone involving apple fruit ripening (Saure, 1990) and also a key regulator of anthocyanin development, because in the apple cultivar ‘Pink Lady’, anthocyanin biosynthesis is significantly correlated with ethylene production (Whale and Singh, 2007). Application of ethylene exogenously promotes the synthesis of anthocyanin in the fruit skin at ripening (Larrigaudiere et al., 1996; Li et al., 2002). Suppression of *MdACO1*, an ethylene biosynthesis gene, caused poor red pigmentation in apple (Johnston et al., 2009). Low temperature suppresses ethylene production, thereby affecting anthocyanin accumulation (Tromp, 1997; Tatsuki et al., 2011).

## Dark Color Relationship With Pollination

**Figure 2:**
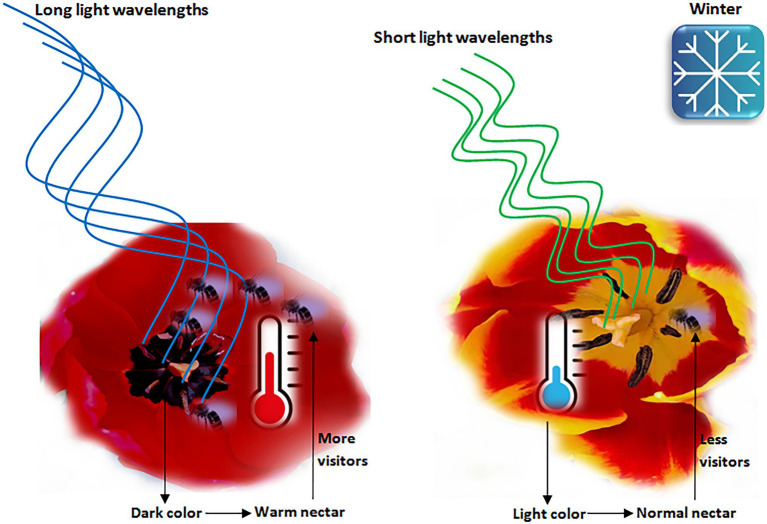
Flower color relationship with heat absorption and pollinators. Dark color flowers absorb sun light with longer wavelengths, which causes increase in the internal temperature of flowers that attract more pollinators in the winter season and allows better pollination. The light color flowers, however, absorb shorter wavelengths of light and do not provide much warm flowers as compared to dark color flowers.

In the drought- and heat-stressed conditions, plants with pigmented flowers can survive much better than anthocyanin-free flowers (Grace and Logan, 2000; Warren and Mackenzie, 2001; Steyn et al., 2002). Arctic flowers with dark colors can reach higher temperatures as compared to light color flowers (Büdel, 1959; Tikhomirov et al., 1960). Petals with dark and deep colors can absorb longer wavelengths of light more efficiently than light color petals, resulting in increased corolla temperature (Tikhomirov et al., 1960; Figure 2).

Pollinators select flowers based on their color attributes such as hue and brightness (Caruso et al., 2010; Koski and Galloway, 2020). It is evident that pollinators contribute to disparities in flower color among populations of the same species (Streisfeld and Kohn, 2007; Sobral et al., 2015; Streinzer et al., 2019). However, differences in pollinator communities are not sufficient to explain variation of color in a number of others (Schemske and Bierzychudek, 2007; Thairu and Brunet, 2015). Therefore, non-pollinator selection agents are also involved in flower color variations (Strauss and Whittall, 2006). Temperature can be a selective factor affecting flower color (Coberly and Rausher, 2003; Lacey and Herr, 2005; Koski and Galloway, 2018). Thus, large scale flower color patterns are associated with climatic gradients (Arista et al., 2013; Koski and Ashman, 2015; Koski and Galloway, 2018). For example, dark color flowers are able to warm reproductive structures more efficiently than light color flowers. They can absorb a lot more solar radiation, thereby increasing their reproductive success in low temperature climates (Lacey et al., 2010). Warm flowers get more pollinator visitation in cold environments (Norgate et al., 2010; Figure 2). Dark colors also favor plant in drought conditions (Warren and Mackenzie, 2001). For example, heavily pigmented *Boechera stricta* are produced in low populations due to increased drought tolerance of pigmented morphs (Vaidya et al., 2018).

Heat absorption provides benefits to both reproductive organs of flowers and pollinators. Basking in the flowers can help insects to elevate their body temperature (Hocking and Sharplin, 1965; Kevan, 1975; Heinrich, 1979; Herrera, 1995). Bees prefer flowers with warm nectar (Dyer et al., 2006) and this choice becomes stronger with decreasing ambient temperature (Norgate et al., 2010). Warm flowers provide high quality reward to pollinators compared with low temperature flowers (Figure 2). For example, sugar production and nectar volume are increased with increasing temperature up to 38°C (Petanidou and Smets, 1996). Dark colored flowers absorb more light and emit it as heat (van der Kooi et al., 2019). Very dark flowers show high intra-floral temperature (Jewell et al., 1994; MCKEE and Richards, 1998; Figure 2). Plants developing at low temperature produce dark color spikes that are up to 2.6°C warmer in full sun (van der Kooi et al., 2019). Anthocyanin pigments regulate flower color plasticity (Lacey and Herr, 2005; Stiles et al., 2007; Anderson et al., 2013). This plasticity in response to temperature exists in most of *Plantago* species (Anderson et al., 2013). The *Lotus corniculatus* flowers with dark keel are 6°C warmer than light-keeled flowers (Jewell et al., 1994). Purple *Ranunculus glacialis* flowers are warmer and make more seeds than white flowers (Ida and Totland, 2014). However, no temperature difference was found in several color polymorphic species (MCKEE and Richards, 1998; Sapir et al., 2006; Mu et al., 2010; Shrestha et al., 2018; Kellenberger et al., 2019).

## Black Anthocyanins

A few flowering and fruit plants with dark-purple to black colors certainly catch consumers’ eyes. They contain very high contents of anthocyanins with high antioxidant activity (Jayaprakasha and Patil, 2007; Kelebek et al., 2008). Blood orange contains activities to reduce cardiovascular risk factors (Paredes-López et al., 2010; Pascual-Teresa et al., 2010), oxidative stress (Bonina et al., 2002) and protect DNA against oxidative damage (Guarnieri et al., 2007). Eye catching purple cauliflowers are potent source of nutrition with health-promoting effects (Chiu et al., 2010). Only a few black anthocyanins have been isolated so far.

Five anthocyanin pigments are identified in blackberries, including cyanidin 3-rutinoside, cyanidin-3-(malonyl) glucoside, cyanidin-3-xyloside, cyanidin 3-glucoside, and cyanidin-3-dioxalyglucoside (Cho et al., 2004; Jordheim et al., 2011). Dark purple color of eggplant is due to anthocyanin nasunin (dalphinidin-3-(p-coumaroylrutinoside)-5-glucoside; Noda et al., 2000; Table 1). Nagai (1921) reported that the accumulation of black pigment in soybean seeds is caused by anthocyanin. Yoshikura and Hamaguchi (1969) identified anthocyanins, cyanidin-3-monoglucoside and delphinidin-3-monoglucoside, responsible for black seeds. The Cyanidin-3-(p-coumaroyl)-diglucosdie-5-glucoside is the most abundant anthocyanin in black carrot, responsible for deep purple color (Nath et al., 2022).

**Table 1:** Major deep color anthocyanins in flowering and food crops.

Crop	Major anthocyanin	Color	References
Blackcurrant	Cyanidin-3-rutinoside	Black	Rubinskiene et al., 2005
Black Berry	Cyanidin-3-glucoside	Black	Hou et al., 2013
Black Rice	Cyanidin-3-glucoside	Black	Hou et al., 2013
Barley	Cyanidin-3-glucoside	Purple	Glagoleva et al., 2022
Eggplant	Dalphinidin-3-(p-coumaroylrutinoside)-5-glucoside)	Dark Purple	Noda et al., 2000
Black carrot	Cyanidin-3-(p-coumaroyl)-diglucosdie-5-glucoside)	Dark Purple	Nath et al., 2022
Soybean	Cyanidin-3-monoglucoside	Black	Yoshikura and Hamaguchi, 1969
Soybean	Delphinidin-3-monoglucoside	Black	Yoshikura and Hamaguchi, 1969
Tulip	Delphinidin-3-glucorhamnoside	Black	Shibata and Ishikura, 1960
Pansy	Delphinidin-5-O-glucoside-3-O-[4-*p*-coumaroylrhamnosyl(1-6)glucosie]	Black	Goto et al., 1978
*Lisanthius nigrescense*	Delphinidin-3-O-rhamnol(1-6)galactoside	Black	Markham et al., 2004
*Cosmos atrosanguineus*	Cyanidin 3-O-glucoside and 3-O-rutinoside	Black	Amamiya and Iwashina, 2016
Dahlia	3-(6″-malonylglucoside)-5-glucoside	Black	Deguchi et al., 2016
Phalaenopsis	Cyanidin	Black	Hsu et al., 2019
Chrysanthemum	Cyanidin	Purple Red	Noda et al., 2000
Chrysanthemum	Delphinidin 3-(3*″,6″*-dimalonyl) glucoside	Violet	Noda et al., 2013
Chrysanthemum	Delphinidin 3-(6″-malonyl) glucoside	Violet	Noda et al., 2013

Higher amount of melanin pigment was found in the black seed coat of rapeseed (Zhang et al., 2008). Black rice is an important health-promoting food due to abundance of anthocyanins and thermal degradation is a major issue to food industry (Hou et al., 2013). Four anthocyanins are identified in black rice, including cyanidin-3-rutinoside, peonidin-3-glucoside, cyanidin-3-glucoside, and cyanidin-3,5-diglucoside. Cyanidin-3-glucoside is a major anthocyanin found in black rice and blackberry (Hou et al., 2013; Figure 1A). In purple barley, the most abundant anthocyanin is cyanidin 3-glucoside (Glagoleva et al., 2022) The black *Phalaenopsis* flowers are important breeding sources to induce color variation in floriculture crops. The harlequin/black *Phalaenopsis* flowers contain black spots on petals, appearing as a new color in 1996 (Chen, 2004; Hsu et al., 2019).

The *Tulipa julia* contains black portion on the lower side of petals and a hybrid tulip ‘Queen of the Night’ contains highly saturated violet color which appears black under specific light conditions (Markham et al., 2004; Figure 2). A study on 107 tulip cultivars identified five selections with black flowers (Shibata and Ishikura, 1960). Delphinidins were the predominant anthocyanins, including delphinidin (50%), cyaniding (29%) and pelargonidin (21%). For black ‘Queen of the Night’ tulip, tulipanin (delphinidin-3-glucorhamnoside) was the most prominent delphinidin glycoside. A *p*-coumroyltriglycoside of delphinidin is responsible for black color of *Viola* cultivar ‘Jet Black’ (Takeda and Hayashi, 1965). Violanin, delphinidin-5-O-glucoside-3-O-[4-*p*-coumaroylrhamnosyl(1-6)glucosie] is the black anthocyanin in the black pansy, *Viola tricolor* (Goto et al., 1978; Table 1). *Lisanthius nigrescense* is unique for its black color corolla (Markham et al., 2004). HPLC analysis showed the presence of one major and one minor anthocyanin. The anthocyanins [delphinidin-3-*O*-rhamnol(1-6)galactoside and its 5-*O*-glucoside] comprised 24% of dry weight of petals. The high anthocyanin level is thought to be responsible for complete absorption of both visible and UV wavebands (Markham et al., 2004).

Two major anthocyanins (cyanidin 3-*O*-glucoside and 3-*O*-rutinoside) were found in the back flowers of *Cosmos atrosanguineus* cultivar ‘Choco Mocha’ (Amamiya and Iwashina, 2016; Table 1). Total anthocyanin contents of black flower cultivars ‘Brown Rouge’ and ‘Choco Mocha’ were 3–4 folds higher than that of red flower cultivar ‘Noel Rouge’ (Amamiya and Iwashina, 2016).

In most black cultivars, high accumulation of cyanidin-based anthocyanins was induced by post-transcriptional silencing of *DvFNS* (*flavone synthase II*) gene (Deguchi et al., 2013, 2015). Cyanidin-based anthocyanins impart more black color to dahlia flowers than pelargonidin-based anthocyanins (Deguchi et al., 2016). The 3-(6″-malonylglucoside)-5-glucoside was the key cyanidin anthocyanin causing black flowers by lowering petal lightness and chroma (Deguchi et al., 2016). Abolishment of competition for substrate between flavone biosynthesis and anthocyanin biosynthesis may be related to increased accumulation of anthocyanin (Deguchi et al., 2016).

The black flower color of dahlia (*Dahlia variabilis*) is caused by high accumulation of cyanidin-based anthocyanins (Deguchi et al., 2013; Table 1). The black dahlia cultivars have strong Type 1 promoter of *DvIVS* with high expression levels (Figure 1C). However, the expression of *DvFNS* was significantly low in all black cultivars. Surprisingly, *DvFNS* suppression occurs in a post-transcriptional manner in black cultivars. Artificial silencing of *FLS* or *FNS* causes increased accumulation of anthocyanins in petunia (Davies et al., 2003). Therefore, silencing of *DvFNS* causing flavone absence abolishes the competition for substrates (Figure 1E). The substrate destined for the synthesis of flavone becomes available for anthocyanidin synthesis. Then using Type 1 promoter of DvIVS helps black cultivars to synthesize high amounts of anthocyanidin from large substrate including the new portion as well, leading to black color appearance (Deguchi et al., 2016; Figure 1E).

## Conclusion and Future Perspectives

Black flower color is very rare in the nature and only a few species produce black flowers. A number of studies tried to justify the causes and benefits of black color in the plants. So far, the studies have found that anthocyanins are the key components of black color accumulation, especially the cyanidin-type of anthocyanins are the most important to drive black color. R2R3-MYB TFs, especially MYB11, are the key regulators of black anthocyanin accumulation in plants. Artificial induction of black color can be achieved through *FNS* silencing, allowing increased synthesis of black anthocyanins using *IVS* promoter. The major benefit of black color is the greenhouse effect it generates by absorbing long wavelengths of light, thereby providing warmth inside petals and warm nectar to attract more pollinators during the winter. Therefore, during extreme winter conditions, deep color of flowers helps plant attract more visitors than light color flowers, that increases the chances of pollination and helps plant survive during harsh conditions.

The best future aspect of black anthocyanins is their stability against temperature extremes and this can be used at industrial level to induce color stability in food products. Moreover, the ornamental flowers with deep color can withstand a long time without color deterioration. Besides, breeding plans can be adjusted for crops growing in extreme winter conditions with difficult pollination breeding. New varieties with greater ability of absorbing long wavelengths of light and deep color flowers would get maximum chances of survival.

## Author Contributions

SA: conceptualization and writing—original draft. JC and GC: data curation and software. JH: investigation. YZ: visualization, investigation, and editing. KZ: data curation and conceptualization. SL: software and editing. ZL: supervision, conceptualization, and funding acquisition. DP: supervision, conceptualization, funding acquisition, and writing—reviewing and editing. All authors contributed to the article and approved the submitted version.

## Funding

This work was supported by National Key Research and Development Program of China (2019YFD1001003).

## Conflict of Interest

The authors declare that the research was conducted in the absence of any commercial or financial relationships that could be construed as a potential conflict of interest.

## Publisher’s Note

All claims expressed in this article are solely those of the authors and do not necessarily represent those of their affiliated organizations, or those of the publisher, the editors and the reviewers. Any product that may be evaluated in this article, or claim that may be made by its manufacturer, is not guaranteed or endorsed by the publisher.
